# Strength surpasses relatedness–queen larva selection in honeybees

**DOI:** 10.1371/journal.pone.0255151

**Published:** 2021-08-05

**Authors:** Saad Naser AL-Kahtani, Kaspar Bienefeld

**Affiliations:** 1 Institute for Bee Research Hohen Neuendorf & Humboldt University Berlin, Hohen Neuendorf, Germany; 2 Department of Arid Land Agriculture, College of Agricultural and Food Sciences, King Faisal University, Al-Ahsa, Kingdom of Saudi Arabia; Universitat Leipzig, GERMANY

## Abstract

Nepotism was initially theoretically predicted and sometimes found to trigger the selection of specific larvae to be reared as queens in the honeybee *Apis mellifera*. Although the importance of selecting the next queen for a colony indicates that it should not occur at random, nepotism is increasingly considered unlikely in eusocial insect societies. Different prenatal maternal supplies of embryos have been found to impact fitness in many other species and therefore could be a possible trigger underlying the likelihood of being raised as a queen. We offered related or unrelated larvae from six colonies originating from eggs of different weights for emergency queen rearing in queenless units with worker bees from these six colonies. We showed that nurses did not significantly prefer related larvae during queen rearing, which confirms the theory that different relatedness-driven kin preferences within a colony cannot be converted into a colony-level decision. However, we found that larvae originating from heavier eggs were significantly preferred for queen breeding. Studies on other species have shown that superior maternal supply is important for later reproductive success. However, we did observe tendencies in the expected direction (e.g., queens that hatched from heavier eggs had both more ovarioles and a shorter preoviposition period). Nevertheless, our data do not allow for a significant conclusion that the selection of larvae from heavy eggs truly offers fitness advantages.

## Introduction

Natural selection favors efficient cooperation within eusocial insect colonies; however, queen rearing may lead to conflicts in polyandrous species [1]. In the honeybee *Apis mellifera* (L.), the mating of queens with an average of 12 haploid males [2] produces colonies with high levels of variation in genetic relatedness. On average, subfamilies of workers that originate from the same drone father (i.e., “supersisters”) share 75% of their alleles, whereas workers of different subfamilies share only 25% of their genes (i.e., “half-sisters”). Workers who display nepotism by raising supersisters instead of half-sisters as new queens may benefit from an increase in their inclusive fitness, at least theoretically [3]. However, studies that provide significant support for nepotism in social insect societies are rare and co ntroversial [4, 5]. Indeed, nepotism is theoretically unlikely because intracolonial nepotism is assumed to decrease the inclusive fitness of all colony members and natural selection should tend to erode cue diversity in genetic recognition systems [6–8]. Nonetheless, Page et al. [9] and Arnold et al. [10] showed that the cuticular hydrocarbon (CHC) profiles of worker honeybees are significantly different between patrilines, a result that has been confirmed for many other insect species [11]. The diversity of cuticular hydrocarbon profiles between patrilines provides a potential mechanism by which workers could act nepotistically. However, most of the data on kin informative odor profiles are from adult individuals, and Breed et al. [12] suggested that young worker bees lack a perceptible chemical profile, which is even more likely to be the case during the very early stage (48 h) when larvae are selected to be reared as queens. Interestingly, several studies [13–17] have shown that *A*. *mellifera* queens are preferentially reared from rare "royal" patrilines. Consequently, they should be recognized as more valuable for queen rearing at this early stage of development or the often-observed bias should result from selection at later stages of queen development (i.e., during the pupal stage after hatching).

Regardless of the mechanisms driving queen rearing, it is extremely unlikely that the larvae are chosen at random. While relatedness is a possible driver of queen rearing behavior by nurse bees, environmentally driven larval preference and/or insufficient heritable variation in recognition cues [18] between the patrilines should also strongly affect the experimental outcomes. Depending on the experimental design and sampling method, nepotism may be either masked or incorrectly proven. Consequently, we tried to avoid these experimental pitfalls by using related vs. nonrelated broods of different genetic origins to provide sufficient heritable variation in recognition cues and rearing the insects artificially under standard laboratory conditions. However, the many unsuccessful attempts to prove that nepotism exists during queen rearing indicates that additional factors that may play a role in the selection of larvae for queen rearing should be investigated.

All social insect societies should be highly invested in female reproductive animals due to their limited number (compared to males) and because the queens typically have long lifespans [19]. Therefore, investigations should focus on the queen’s phenotype and genotype as well as environmental impacts that are likely to affect reproductive success [20]. Maternal investment in the development of offspring has a profound impact on the survival and future reproductive success of the young [21]. For example, Wei et al. [22] found that eggs were heavier in queen cells than in worker cells and that this different maternal investment influences gene expression and adult queen morphology. Many other studies have also found that prenatal care has a significant impact on the fitness of offspring [23]. Not all eggs laid by the queen in queen cells are used by worker bees for queen rearing, and although these eggs are heavier on average, they vary in weight [24]. Moreover, emergency queen rearing, which occurs after the sudden death of the queen and involves the worker bees immediately selecting young larvae to rear as a new queen, is likely to offer even greater prenatal maternal variation. We were interested in whether different prenatal maternal supplies also influence the decision of worker bees to accept larvae for queen rearing.

## Materials and methods

### Observations of preference based on relatedness and maternal investment in honeybee larvae

#### Honeybee colonies

This study was conducted at the apiary of the Institute for Bee Research, Hohen Neuendorf (Germany). Institutional ethics committee of Institute for Bee Research Hohen Neuendorf & Humboldt University Berlin, Hohen Neuendorf, Germany approved this study. To generate sufficient genetic diversity within our samples, we sampled queens from various regions in Germany and Austria and inseminated them with semen from ten drones, each of which had different unrelated origins. Altogether, six honeybee colonies were each divided into a queenless rearing unit (i.e., the larvae-receiving colony, or LRC) and a queenright unit to produce eggs (i.e., the egg-producing colony, or EPC) to be transferred into the LRC for possible queen rearing.

### Rearing of eggs and larvae

**Fig 1:**
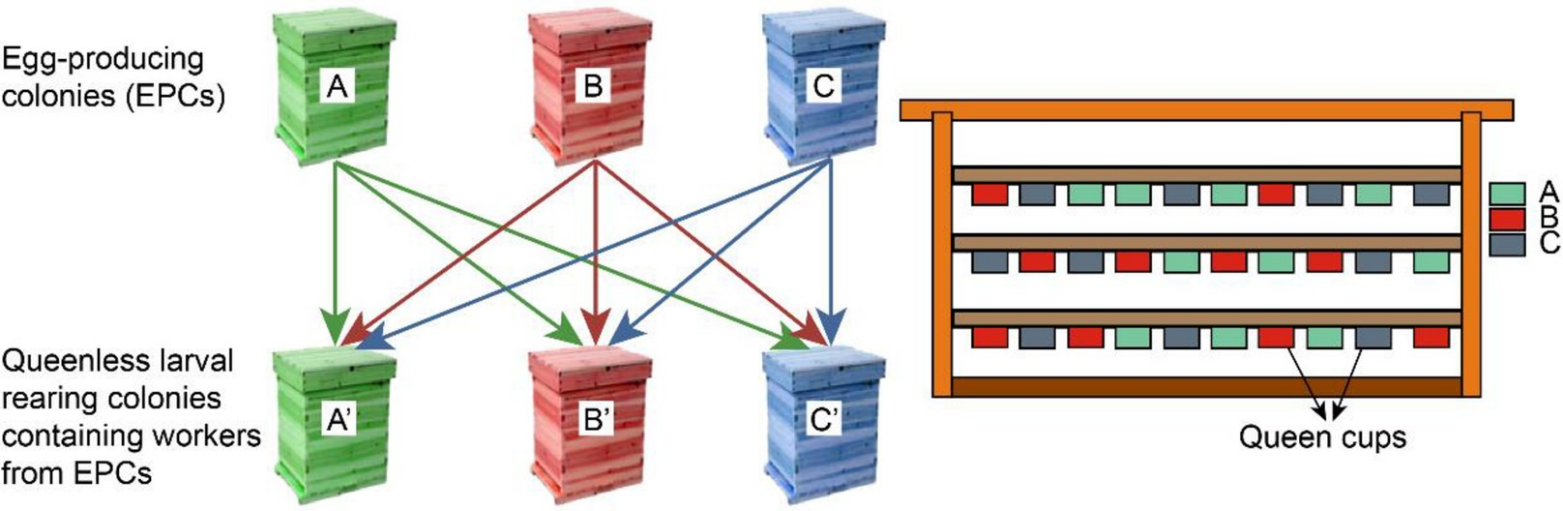
Illustration of experimental design used in the study.

Because the egg weight changes during development until the larvae hatch approximately three days after being laid by a queen [25], eggs of known age were obtained by confining each queen for 6 h on a comb with empty brood cells. The newly laid eggs were transferred to an incubator (35 ± 1°C, 85% relative humidity), and at a standard age of 48 ± 3 h after egg laying, all of the eggs were weighed (Sartorius M5P) to the nearest microgram. To avoid possible artifacts related to different non-genetic colony odors (e.g., a different nectar and/or pollen supply of the colonies), the larvae were artificially reared until an age of 48 h [25]. At this age, 450 larvae, with 30 from each EPC, were transferred into artificial queen cups and distributed equally in the corresponding LRCs. In each of the experiments, each LRC received 10 (related) larvae from its related EPC and 10 larvae from each of the two unrelated EPCs (Fig 1, Table 1). This design was repeated twice using the same EPC × LRC combinations in the first year. In the second year, the experiments were repeated three times (see Table 1 for details). The larvae that were not accepted for queen rearing were removed by the worker bees. The acceptance of larvae for queen rearing could be clearly observed based on the capping of the corresponding queen cells.

**Table 1:** Distribution of larvae from the Egg Producing Colonies (EPC) between the Larvae Receiving Colonies (LRC).

Year	Number of Repetitions	EPC	LRC						Total
			A	B	C	D	E	F	
1	2	A	20	20	20				60
1	2	B	20	20	20				60
1	2	C	20	20	20				60
2	3	D				30	30	30	90
2	3	E				30	30	30	90
2	3	F				30	30	30	90
Total			60	60	60	90	90	90	450

### Quantification of queen traits

Once the queen cell cups were capped, the queen cells were placed in individual plastic vials and stored in an incubator (33 ± 1°C and 60–65% relative humidity) until emergence. The 248 newly emerged queens were weighed (Sartorius M5P) to the nearest microgram and placed in mating nuclei [26]. The virgin queens were allowed to mate naturally. After mating, the preoviposition period (time between emerging and starting egg laying) (*n* = 190) was recorded. A total of 147 queens were dissected 60 days after oviposition, and the number of ovarioles in the left and right ovaries was counted [27].

#### Statistical analysis

A generalized linear mixed model (GLMM) with a binary distribution and a logit link function was used to test for possible differences in the probability that a larvae was accepted (1) or not accepted (0) fur queen rearing. The factors LRC (larvae-receiving colony), EPC (egg-producing colony) and relatedness (yes or no) were considered fixed effects. Egg weight was considered a continuous variable. The repetitions of the experiments were set as a random effect. The weights (at 48 h) of eggs that developed into the larvae that were ultimately selected for queen rearing as well as the weight of eggs that did not produce larvae selected for queen rearing were subjected to an ANOVA. To investigate whether larvae of individual EPCs were selected disproportionately for the rearing of queens, regardless of the relationship between EPCs and LRCs, we also evaluated the influence of maternal ancestry (Chi2 test) and whether possible differences were associated with the average egg weight of the queens of the EPCs. For this, we used a correlation analysis between the acceptance of larvae of EPCs for queen rearing and the respective average weight of the eggs at the time of the respective experiments. To determine the impact of egg weight on queen weight, the ovariole number and preoviposition period were estimated by regression, while the queen traits were adjusted for the effects of the experiment number and the LRC nested within the experiment number.

## Results

### Observations of preference based on relatedness and maternal investment in honeybee (*Apis m*. *carnica*) larvae

We compared the proportions of related (same colony) and unrelated (alien colony) reared larvae (48 h) that were selected for queen rearing across six queenless colonies. No significant preference for rearing-related larvae, such as queens, was found (F_1,435_ = 0.51, P> 0.48). The factor EPC (egg producing colony) did not significantly affect queen rearing (F_4,435_ = 0.40, P = 0.81), but the nursing colony (LRC) (F_4,435_ = 3.30, P = 0.01) did significantly impact the amount of queen rearing. The factor that was most important for preferential royal treatment was the weight of the egg from which the larvae emerged (F_1,435_ = 13.3, P = 0.0003).

The average weight of the eggs (from which the larvae originated) was significantly heavier (ANOVA, *n* = 450, F_1-448_ = 22.23, *P* = 0.0001) of the larvae that were successfully accepted for queen rearing (0.163 ± 0.23 mg, *n* = 248) than the average egg weight of larvae not accepted for queen rearing (0.153 ± 0.22 mg, *n* = 202).

We found no significant difference in the preference of larvae of individual mothers for the rearing of queens (Table 2), and the average weight was not significantly different between the queens (F = 1.00, p = 0.44); however, we found significant differences between the experiments regarding the average egg weight of queens. (F = 5.04, p = 0.0006). Consequently, we compared the results of the average acceptance of larvae of the individual EPCs with the average egg weight of these queens at the time of each experiment (SI 1). The significant correlation (r = 0.55, p = 0.032, N = 15) between the two parameters indicates that queens with a higher average egg weight have a greater chance that their larvae will receive royal treatment compared to queens with a lower average egg weight.

**Table 2:** Mean and standard deviation of egg weight per EPC (egg producing queen) and acceptance of the larvae of these EPQ for queen rearing.

EPC	Egg weight			% of accepted larvae
	N	X	Std	
A	60	162.7	22.2	60.0
B	60	157.7	23.6	50.0
C	60	154.9	25.1	48.3
D	90	155.8	20.2	53.3
E	90	161.2	22.8	58.9
F	90	157.6	21.5	57.8
Total	450	158.3	22.8	55.1
Significance Level	F = 1.25, p = 0.28			Chi^2^ = 3.22, p = 0.67

To determine whether stronger prenatal provision has a positive impact on the later phenotype of the developed queens, we correlated egg weight with various queen characteristics that supposedly influence reproduction. A significant relationship was not observed between better prenatal provisions (represented by egg weight) and any of the adult queens’ three recorded characteristics: body weight (coefficient of regression: b = 45.59 ± 86.78, *P* = 0.59, *n* = 248), number of ovarioles (b = 115.77 ± 195.28, *P* = 0.55, *n* = 147), and length of the preoviposition period (b = –11.40 ± 17.30, *P* = 0.51, *n* = 190).

## Discussion

### What influences the acceptance of larvae for queen rearing in a colony?

This study was designed to prevent false indications of nepotism by using standardized artificially reared larvae and to enable possible nepotism indications by ensuring genetic diversity among the patrilines available. We found no significant preference for the rearing of related larvae. If kinship does truly play a role in the selection of larvae for queen rearing, it should have been clearly indicated using the traditional experimental approach applied here. Compared with the natural conditions under which worker bees must differentiate between the more closely or distantly related offspring larvae of a single mother, only the differentiation between related and unrelated larvae is necessary for this study design. The fact that this essential difference with not observed in this study or in many others involving both honey bees and other social insects contradicts the hypothesis that kinship plays a central role in the selection of larvae for queen breeding in eusocial societies [3–5].

Sagili et al. [28] showed that nutritional status has an impact on the likelihood of young larvae being selected for queen rearing. Our data showed that even at an earlier stage in larval development, superior prenatal provisioning (egg weight) significantly increased this probability, with larvae from heavier eggs significantly more likely to be reared as queens. The size of the embryo shortly before hatching is strongly correlated (r > 0.9) with egg size [29]; thus, this parameter should be used for estimating the impact of the prenatal environment on larval development. However, the average differences in egg weight between the two groups (accepted for queen rearing or not) was significant but small. We speculate that this small prenatal benefit initiates better postnatal care from nurse bees and/or epigenetic effects, which then (> 24 h after hatching) result in a higher probability of royal treatment. Insufficient prenatal nutrition has been found to have lasting reproductive constraints throughout the reproductive lives of many species [30].

If prenatal provisions have the same positive effect on the fitness of honeybees as demonstrated for reproducing females of other species, then this selection criterion will have a decisive advantage because it is patriline neutral and therefore independent of the genetic composition of the colony, which serves the fitness of all colony members. The extremely high weight variability among simultaneously laid eggs [26] provides a good basis for offering some individual larvae exceptionally good starting conditions. Different pre- and postnatal effects may affect the size and other morphometric criteria of the larvae, which could then influence the chances that the larvae will be selected as queens. Differential postnatal feeding of newly hatched honeybee larvae was found to modify DNA methylation patterns, which had significant implications for the adult phenotype [31]. While prenatal maternal effects have been found to significantly affect the epigenetic regulation of genes in other species [32], limited evidence supports the epigenetic effects of prenatal maternal investment on honeybee larval phenotype (e.g., CHC profile) in terms of its attractiveness for queen rearing. However, variations in CHC profiles and different sexual attractiveness due to dietary changes have been reported for males of *Drosophila simulant* [33].

Is there a similar “silver spoon effect” [34] among honeybees, in which above-average prenatal provisions lead to better reproductive phenotypes in adult queens? Borodacheva [35] observed a significant positive correlation between the size of the egg from which the queen originated and the queen’s number of ovarioles. Additionally, Wei et al. [22] showed that heavier eggs selectively laid by queens in queen cells resulted in significantly heavier queen offspring. In contrast, significant individual relationships were not observed between egg weight and any of the traits used to describe reproductive potential (e.g., body size, number of ovarioles, and pre-oviposition period of queens).

However, we did observe tendencies in the expected direction (e.g., queens that hatched from heavier eggs had both more ovarioles and a shorter pre-oviposition period). Nevertheless, our data do not provide strong support for the conclusion that the selection of larvae from heavy eggs truly offers fitness advantages. It should be emphasized, however, that the static proof in this case causes methodological difficulties. Due to the preferential raising of larvae from heavier eggs, hardly any data were obtained for larvae from light eggs, thereby resulting in a biased sample. These non-representative available data may have led to an underestimation of the actual relationship between these characteristics.

### Do bees react to genetic differences when selecting larvae for queen rearing?

Analyses based on allozymes or DNA markers [13–17] have identified a bias toward selecting individuals for queen rearing from particular patrilines compared to the relative occurrence of these individuals in the analyzed worker samples. All these studies present similar conclusions that a significant preference occurs for individuals from particular “royal” patrilines. In our study, we could not confirm that larvae from different queens with genetic differences (which likely had greater genetic differences than that between patrilines within a colony) received significantly different non-nepotism-based preferences for queen rearing. However, we found that an environmental influence on the average weight of the eggs of queens can affect the chance of producing more or less royal offspring. The correlation between the two measurements was significant at r = 0.55.

Our results can explain why the above-average prenatal supply among a queen’s offspring simulates higher genetic attractiveness of the larvae for queen production, and consequently that environmental driven advantages are then be interpreted as more genetically attractive.

However, this interpretation does not apply to the observed [13–17] disproportionate occurrence of individual patrilines in queen production within a colony. In this case, it is unrealistic to assume that the different attractiveness of individual patrilines (rare royal patrilines) is caused by targeting a better prenatal supply for representatives of individual patrilines within a queen. Consequently, in this case, the significant preference of individuals from particular patrilines is likely to be caused by their different genotypes. It should be mentioned that the proof for this preference was shown in the bias toward selecting individuals from particular patrilines compared to their relative occurrence in the analyzed worker samples of colonies. This approach is extremely prone to experimental flaws in the sampling of workers and queens, especially if the number of patrilines per colony is high. However, if genetic differences between patrilinies lead to different preferences for queen breeding, then the influence of the genotype on the cuticular hydrocarbon (CHC) profiles could be a possible explanation. CHCs are involved in various recognition functions in social insects [36]. Page et al. [9] and Arnold et al. [10] showed that adult worker bees of different patrilines have different CHC profiles, and evidence has shown that CHC profiles also differ in honey bee larvae [37] and are not only used for recognition in certain species but can also convey information about individual fertility [38–41]. Whether different patrilineal preferences are caused by different CHC profiles and whether "more attractive" CHCs are truly associated with fitness advantages have not been determined. If these characteristics do not offer any selection advantages for the emerging queens, then they are only used for cheating [42] and do not provide sustainable advantages for the selection unit, i.e., the colony. Although the case is not directly comparable Jordan et al. [43] showed that a high percentage of new queens of the thelytokous South African honeybee subspecies *Apis mellifera capensis* originated from thelytokously reproducing parasitic *A*. *m*. *capensis* worker bees from other colonies. Moreover, the effect of the genotype of the larvae on the attractiveness for queen breeding is supported by Beekman et al. [44], who found that larvae from *A*. *m*. *capensis* received preferential royal treatment from European honeybee workers. Both are examples of the combination of *A*. *m*. *capensis* and cheating. To the best of our knowledge, no corresponding evidence could be found in the other arrhenotokous subspecies of *A*. *mellifera*. In eusocial polyandrous species, where all colony members are more or less related, the costs of kin differentiation are likely to exceed the benefits if it has any benefit at all; however, this situation is very different when the attractiveness for queen breeding is caused by a better prenatal supply. As shown in many studies in other animal species, favorable conditions in the early stages of development influence later fitness [29]. The increased environmental fitness of the new queen is an advantage for all members of the bee colony. A within-colony selection advantage caused by genetic differences (e.g., CHC profiles) without between-colony fitness advantages is likely to be disadvantageous for the colony as a whole.

## Conclusion

Honeybee larvae are not selected at random for gyne production, and although genetic relatedness has no effect, prenatal environmental exposure does impact the likelihood that a larva will be reared as a queen. Although evidence from other studies has supported the influence of different larval genetics on a preference for queen breeding, such an influence was not observed here. However, we do not want to rule out this option in principle. Further research is required to determine the factors that trigger the royal succession decision by worker bees in colonies and identify how the possible drivers (prenatal environment and genetic makeup of the larvae) interact to maximize fitness advantages.

## Supporting information

S1 Table Average egg weight of EPC (egg producing colonies) and the acceptance of the larvae of these EPQ for queen rearing at the totally 15 observations. (DOC)
