# Supplementary material for: Strength surpasses relatedness–queen larva selection in honeybees
Source: PLoS One. 2021 Aug 5;16(8):e0255151. doi: 10.1371/journal.pone.0255151 (PMC8341480; doi:10.1371/journal.pone.0255151)
Supplement: S1 Table — (DOC) [file pone.0255151.s001.doc]

**Strength surpasses relatedness – queen larva selection in honeybees**

Saad Naser AL-Kahtani^1,2^,*, Kaspar Bienefeld^1^*

^1^Institute for Bee Research Hohen Neuendorf & Humboldt University Berlin, Hohen Neuendorf, Germany

^2^Department of Arid Land Agriculture, College of Agricultural and Food Sciences, King Faisal University, Al-Ahsa, Kingdom of Saudi Arabia

*Correspondence: salkahtani@kfu.edu.sa (SNA); [kaspar.bienefeld@hu-berlin.de](mailto:kaspar.bienefeld@hu-berlin.de) (KB)

**SUPPORTING INFORMATION**

**Table S1:** Average egg weight of EPC (egg producing colonies) and the acceptance of the larvae of these EPQ for queen rearing at the totally 15 observations

| EPC | Exp. No. | Larvae total | Larvae accepted | Average eggs accepted (%) | weight (mg) |
| --- | --- | --- | --- | --- | --- |
| A | 1.1 | 30 | 21 | 70.0 | 0.165 |
| A | 1.2 | 30 | 15 | 50.0 | 0.161 |
| B | 1.1 | 30 | 17 | 56.7 | 0.163 |
| B | 1.2 | 30 | 13 | 43.3 | 0.152 |
| C | 1.1 | 30 | 17 | 56.7 | 0.159 |
| C | 1.2 | 30 | 12 | 40.0 | 0.150 |
| D | 2.1 | 30 | 10 | 33.3 | 0.154 |
| D | 2.2 | 30 | 23 | 76.7 | 0.166 |
| D | 2.3 | 30 | 15 | 50.0 | 0.147 |
| E | 2.1 | 30 | 15 | 50.0 | 0.161 |
| E | 2.2 | 30 | 20 | 66.7 | 0.164 |
| E | 2.3 | 30 | 18 | 60.0 | 0.158 |
| F | 2.1 | 30 | 10 | 33.3 | 0.159 |
| F | 2.2 | 30 | 23 | 76.7 | 0.163 |
| F | 2.3 | 30 | 19 | 63.3 | 0.150 |
